# Capsaicin: From Plants to a Cancer-Suppressing Agent

**DOI:** 10.3390/molecules21080931

**Published:** 2016-07-27

**Authors:** Angela M. Chapa-Oliver, Laura Mejía-Teniente

**Affiliations:** 1 Departamento de Nutrición, Universidad de Celaya, Carretera Panamericana km. 269 Col. Rancho Pinto, Celaya 38080, Mexico; angela.chapa@udec.edu.mx; 2 C.A. Biotecnología, Sustentabilidad e Ingeniería, Programa de Ingeniería en Biotecnología, Departamento de Ingeniería Agroindustrial, División de Ciencias de la Salud e Ingenierías, Campus Celaya-Salvatierra, Universidad de Guanajuato, Av. Mutualismo Esq. Prolongación Río Lerma S/N, Celaya, Gto. C.P. 38060, Mexico

**Keywords:** capsaicin, elicitors, stress, cancer, apoptosis, cell death

## Abstract

Capsaicinoids are plant secondary metabolites, capsaicin being the principal responsible for the pungency of chili peppers. It is biosynthesized through two pathways involved in phenylpropanoid and fatty acid metabolism. Plant capsaicin concentration is mainly affected by genetic, environmental and crop management factors. However, its synthesis can be enhanced by the use of elicitors. Capsaicin is employed as food additive and in pharmaceutical applications. Additionally, it has been found that capsaicin can act as a cancer preventive agent and shows wide applications against various types of cancer. This review is an approach in contextualizing the use of controlled stress on the plant to increase the content of capsaicin, highlighting its synthesis and its potential use as anticancer agent.

## 1. Introduction

Pepper is a member of the Solanaceae family and is one of the oldest domesticated crops in the Western Hemisphere [1,2]. The importance of this crop is that consumption continues to grow because of its high nutritional value, since it is a rich source of vitamins C and E as well as provitamin A and carotenoids, compounds with well-known antioxidant properties [1,3]. Peppers are mainly consumed as food additives because of their unique pungency, aroma, and color. Only in 2011, the value of global hot pepper production was $14.4 billion, 40-fold higher than in 1980 [1].

The genus *Capsicum* consists of approximately 25 wild and five domesticated species. The five domesticated species are *Capsicum annuum*, *C. baccatum*, *C. chinense*, *C. frutescens*, and *C. pubescens*. Of the domesticated species, *C. chinense* is the most pungent fruit type [4]. The pungent principles of chili fruit are capsaicinoids, which are alkaloids that accumulate in the placenta of maturing *Capsicum* (chile pepper pods), and have wide applications in food, medicine, and pharmacy. Around 80%–90% of capsaicinoids present in *Capsicum* consist of capsaicin and dihydrocapsaicin, in a ratio of approximately 1:1 and 2:1, respectively. The rest is comprised of nordihydrocapsaicin, homodihydrocapsaicin, homocapsaicin, norcapsaicin, and nornorcapsaicin [5,6,7].

**Figure 1:**
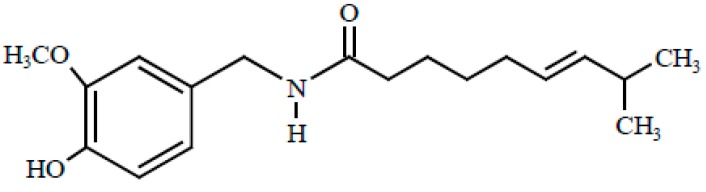
Chemical structure of capsaicin [7].

Capsaicin (*trans*-8-methyl-*N*-vanillyl-6-nonenamide) is a homovanillic acid derivative responsible for the characteristic pungent feeling of the genus *Capsicum* (Figure 1) [8]. It has been found that a change in the acid portion of capsaicin generates analogs with different degrees of pungency [9]. Castillo et al. [10] obtained three analogs of capsaicin, two pungent and one with very low pungency. To achieve this, they used different acyl chain lengths and chemical substitutes in the aromatic ring [10]. Additionally, this metabolite concentration, like other plant secondary metabolites, can be risen under controlled stress [11]. Both capsaicin and its analogs have been used medicinally for centuries, but recently it has been extensively studied for its analgesic, antioxidant, anti-inflammatory, and anti-obesity properties, and, most recently, its anticancer activity against a variety of cancer types [12,13,14,15,16,17,18].

Therefore, the aim of this review is to contextualize the use of controlled stress on the plant to increase the content of secondary metabolites, highlighting the synthesis of capsaicinoids and their potential use as anticancer agents.

## 2. Crops with Higher Content of Capsaicin

The primary capsaicinoids, capsaicin, dihydrocapsaicin and nordihydrocapsaicin, are produced exclusively in the epidermal cells of the chili placenta. Within the domesticated species of the genus *Capsicum*, *Capsicum chinense* is recognized as having the most pungent fruit [19].

The pungency level is expressed in Scoville Heat Units (HUS), which is an arbitrary unit and depends on the palate of the taster. It was developed by Scoville in 1912, and is related to the amount of capsaicin present in the sample. It is known that the human palate can detect it, even in as diluted a ratio as 1:17,000,000. According to this scale, *C. chinense* Jacq.cv. Naga King Chili, an important pepper crop of India, has been acknowledged as the hottest chili in the world, measuring 1,001,304 SHU [4]. Diverse cultivars of Habanero pepper have also occupied the highest values of this Scoville scale. As more pungent cultivars have become known (Red Savina Habanero 580,000 SHU), the number of levels in the scale has changed. The *C. chinense* species continues to occupy the highest places of the scale [19].

Recently, in a comparative study of pungency between the three most pungent cultivars reported (orange criolle Habanero, Red Savina Habanero, and the variety Bhut Jolokia), all of which belong to the species *Capsicum chinense*, Bosland and Baral [20] found that Bhut Jolokia was significantly more pungent (1,001,304 SHU), making it the most pungent chili pepper known to date, only surpassed by pure capsaicin (16,000,000 SHU), whereas the variety Red Savina Habanero, despite expectations to the contrary, registered a pungency lower than that of the standard orange Habanero pepper (248,556 versus 357,729 SHU) [19]. It is important to note that the Scoville organic test is a subjective measure of chili pungency invented by Scoville in 1912 and since its publication, the cultivars of Habanero pepper have occupied the highest values of this scale [20].

## 3. Capsaicin Biosynthesis

**Figure 2:**
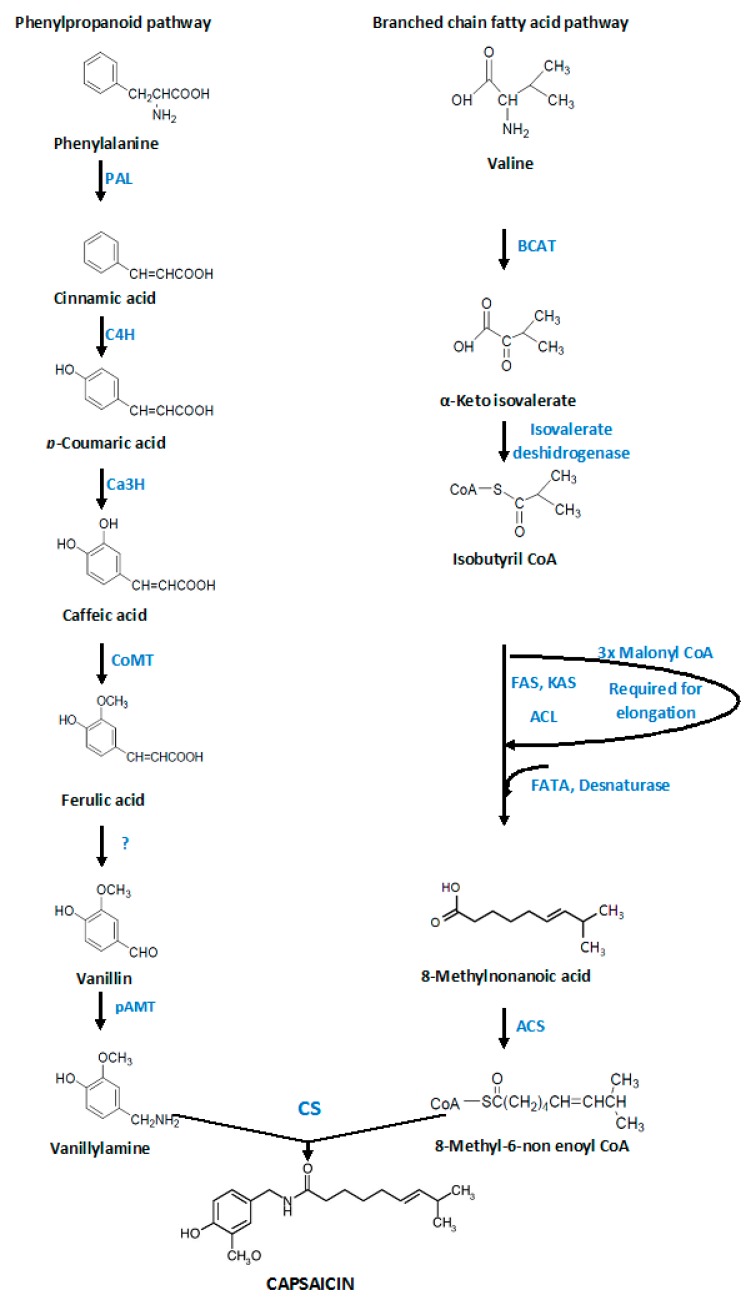
Capsaicin biosynthetic pathway modified from Arora et al. [11]. Enzymes involved in the biosynthetic pathway: PAL, phenylalanine ammonia lyase; C4H, cinnamic acid 4-hydroxylase; Ca3H, coumaric acid 3-hydroxylase; COMT, caffeic acid *O*-methyltransferase; pAMT, putative aminotransferase; BCAT, branchedchain amino acid transferase; FAS fatty acid synthase complex, KAS, β-ketoacyl-[acyl-carrier-protein] (ACP) synthase; ACL, acyl carrier protein; FATA, acyl-ACP thioesterase; Desnaturase; ACS, acyl-CoA synthase; CS, capsaicin synthase.

Despite the proposed biosynthetic pathway for capsaicinoid synthesis was presented by Bennett and Kirby [21] and Leete and Louden [22], many enzymes involved in capsaicinoid biosynthesis are not yet well characterized, and regulation of the pathway is not fully understood. It is now known that there are two main routes for the synthesis of capsaicinoids. The first dependent on the route of the phenylpropanoid, where l-phenylalanine derivatives as cinnamic, p-coumaric, caffeic and ferulic acid and vanillin lead to formation of vanillylamine for the subsequent condensation of capsaicin through capsaicin synthase. The second route involves the metabolism of branched fatty acids, mainly derived from valine or leucine, ending in the formation of 8-methyl-6-nonenoyl-CoA. Finally, capsaicin is generated through a condensation reaction between vainillilamina and 8-methyl-6-nonenoyl-CoA catalyzed by the coenzyme A-dependent acyltransferase (Figure 2) [23,24].

Significant efforts have been made toward understanding biosynthetic pathway of capsaicin. Examples of this are the genes codified for the enzymes phenylalanine ammonia-lyase (PAL), cinnamic acid 4-hydroxylase (C4H), caffeic acid O-methyltransferase (COMT), a putative aminotransferase (pAMT) and a β-keto-acyl-[acyl-carrier-protein] synthase (KAS) that were identified in the cDNA library obtained from placenta tissue of highly pungent peppers (*Capsicum chinense* cv. Habanero) obtained by Curry et al. [7]. A key step in capsaicin biosynthesis is the conversion of vanillin to vanillylamine, and a putative aminotransferase (pAMT) has been proposed to be the enzyme responsible for vanillins transamination. Abraham-Juárez et al. [24] showed that silencing *pAMT* gene through virus-induced gene silencing (VIGS) reduces the level of capsaicin in *C. annuum* L. cv. Tampiqueño, classified as mildly pungent, thus proving that *pAMT* gene was involved in capsaicinoid synthesis. Additionally, Tanaka et al. [25] showed that a single amino acid substitution in AMT protein sequence led to a functional loss.

Several studies have shown that the expression profile of *Pun1* gene, coding for a putative capsaicin synthase (CS), correlates with pepper pungency. Ogawa et al. [23], by silencing the *Pun1* gene in pepper plants, found that the capsaicin levels in placenta tissues were low, compared with control plants, thus demonstrating that *Pun1* gene plays an active role in the synthesis of capsaicin [23]. In addition, it has been reported that *Pun1* has a role in the capsinoids synthesis. Capsinoids are capsaicinoid-like substances that lack pungency and possess similar physiological functions to capsaicinoids. They are present in sweet chili varieties [26,27,28]. Therefore, they turn out to be promising compounds with similar applications of capsaicin. However, sequencing the chili genome opens a key opportunity to gain a complete and clear understanding of the capsaicinoid pathway and represents an excellent resource for exploring the evolution of secondary metabolites in plants [1]. The study of the hot pepper genome sequence suggests that the pungency has its origin in the evolution of new genes by unequal duplication of existing genes and owing to changes in gene expression in fruits after speciation [1].

## 4. Elicitor Induction of Capsaicinoids

Plants are frequently exposed to different environmental stresses types, both biotic and abiotic. These generate biochemical and metabolic changes, resulting in the production of hydrogen peroxide (H_2_O_2_), and reactive oxygen species (ROS). The oxidative burst generated corresponds to the primary response of the defense mechanism in plants [29,30]. However, this signaling cascade can also be activated by the use of elicitors, stable molecules that induce an immune defense response in plants and accumulation of diverse secondary metabolites as part of the defense responses to pathogen infection and environmental stress [30,31].

Elicitors can play an important role in the achievement of long-term crop productivity. Through the use of elicitors during growth, phytonutrients content can be maximized while environmental impacts are reduced [32]. Vargas-Hernández et al. [31] confirmed significant increase of secondary metabolites accumulation such as total phenolics, flavonoids, and capsaicinoids contents, in varieties *C. chinense* treated with H_2_O_2_ compared to control plants. Additionally, they observed an increased in antimicrobial activity in methanolic extracts from plants previously elicited. Effect of Laminarin spray in *C. frutescens* capsaicinoids levels was tested by Gururaj et al. [33]. Their study showed that both vanillylamine, a precursor for all capsaicinoids, and capsaicin levels were nine fold (44.57 ± 1.22 μmol/g dry weight) and 1.7 folds (62.65 ± 1.72 μmol/g dry weight) higher than levels in control.

In addition to the direct effect that the elicitors have on capsaicin accumulation in plants, it has been found that this effect is also achieved in plant cells culture. It was found that root cultures of *Capsicum frutescens*, treated with veratraldehyde, a derivative of vanillin, accumulated more vanillin (78 μM) than caffeic acid fed cultures [34]. Additionally, they observed that efficiencies of biotransformation, respecting to vanillin formation, with caffeic acid and veratraldehyde were 2.2% and 9%, respectively, indicating a possible diversion of the phenylpropanoid pathway towards other secondary metabolites [34]. Islek, et al. [35] studied the effect of different cellulase concentrations on the production of capsaicin in freely suspended and immobilized cell cultures of Kahramanmaraş pepper seeds (*C. annuum* L.). They found that the immobilization process had an increasing effect on the capsaicin accumulation and that the highest capsaicin concentration for the immobilized cells was 362.91 μg/g fresh weight after 24 h.

## 5. Anticancer Activity

There is persuasive epidemiological and experimental evidence that dietary phytochemical found in fruits, vegetables, whole grains, spices and teas exhibit diverse inhibitory effects against cancer initiation, promotion, progression and metastasis [36]. Between them is capsaicin, a bioactive phytochemical abundant in chili peppers. Capsaicin is a homovanillic acid derivative and it has been shown to alter the expression of several genes involved in cancer cell survival, growth arrest, angiogenesis and metastasis [17,18,37,38].

Tumorigenesis is a multi-stage process that generally occurs over an extended period of time. Cancer cells acquire unique capabilities that most healthy cells do not possess [18]. Cancer is initiated and progresses by multiple genetic alterations and aberrant signaling pathways. Identification of molecular targets involved in the steps of tumor development will provide opportunities to establish a promising strategy to fight against cancer [18]. Studies evaluating the capsaicin effect on cancerous cells are showed in Table 1, where displayed the capsaicin effect to inhibit cell proliferation in many cancer cell types by mechanisms that are not completely understood [39]. The proposed anticancer mechanisms of capsaicin include an increase of cell-cycle arrest and apoptosis [18].

## 6. Capsaicin and Apoptosis

**Figure 3:**
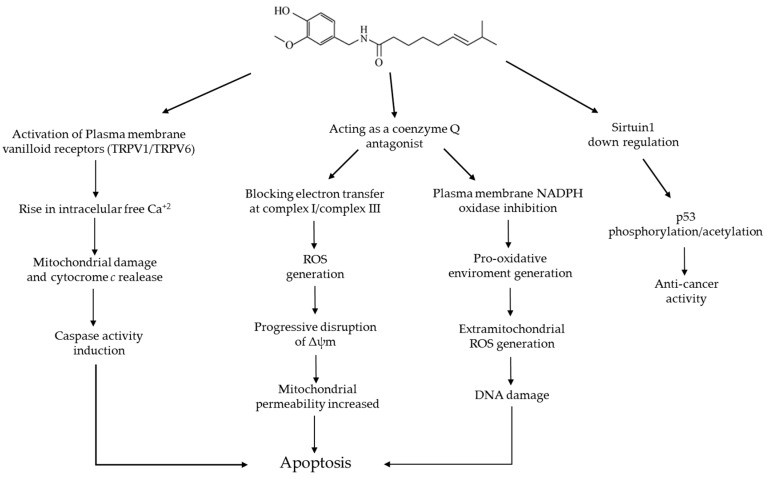
Summary of some of the pro-apoptotic mechanisms and anti-cancer activity of capsaicin in some cancerous cells [17,52,62,72,73,74].

Apoptosis is an essential barrier against cancer development and progression and loss of apoptotic signaling is highly associated with malignancy [37]. It has been recently demonstrated that capsaicin induces apoptosis in many types of cancer cell lines, including colon adenocarcinoma, pancreatic cancer, hepatocellular carcinoma, prostate cancer, breast cancer, and many others, leaving normal cells unharmed [17]. Nevertheless, the molecular mechanism whereby capsaicin induces apoptosis in cancer cells is not completely elucidated but involves intracellular calcium increase, ROS, disruption of mitochondrial membrane transition potential, and activation of transcription factors such as NFκB and STATS (Figure 3) [69,70,71].

It has been found that pro-apoptotic activity of capsaicin is mediated via TRPV1 (transient receptor potential vanilloid type-1) in many types of cancers [75]. TRPV1 is a nonselective cation channel that belongs to the family of transient potential receptors (TRPs) [70]. It prefers Ca^2+^ over Na^+^. Thus, it contributes to changes in cytosolic free-Ca^2+^ concentration and is the primary cellular target of capsaicin [75]. TRPV1 is also broadly distributed in tissues of the brain; bladder; kidneys; intestines; keratinocytes of epidermis, glial cells, liver, and polymorphonuclear granulocytes; mast cells; and macrophages [76]. Until now, capsaicin has been identified uniquely as an agonist of TRPV1. The expression of TRPV1 has been demonstrated in most of the tumor cells analyzed. TRPV1 is expressed in human breast cancer MCF-7 and BT-20 cells [45,77], prostate cancer derived LNCaP and PC-3 cells, and Benign Prostate Hyperplasia (BPH) tissue expresses TRPV1 [51].

Capsaicin has been show to inhibit growth or cause apoptosis in prostate tumor cells, both in vitro and in vivo (Table 2). TRPV1 expression is upregulated in prostate cancer, but it is not the only vanilloid receptor that is related. It has been found that TRPV6 is upregulated in advanced prostate cancer. Compared with normal tissue or cells, the expression of TRPV6 mRNA and expression of the TRPV6 protein is substantially increased in prostate cancer tissue [78]. Its expression is also regulated by the androgen receptor, which highlights the role of TRPV channels in the growth of prostate cells [69,78]. Experiments using capsaicin at 10 μM and above resulted in proliferation reduction of the androgen-independent PC-3 and DU-145 cells and caused apoptosis in vitro [51]. Capsaicin treatment also induced apoptosis in prostate cells by dissipation of the mitochondrial inner transmembrane potential and activation of caspase 3 [51]. The same mechanism of action has been observed in human colon [79] and pancreatic cancer cells [80] treated with capsaicin.

Caprodossi et al. [75] found that capsaicin treatment induced a more aggressive gene phenotype and invasiveness in null-TRPV1 urothelial cancer cells [75]. However, transfection of TRPV1 cDNA in these cells restores the sensitivity of capsaicin induced apoptosis and inhibited the acquisition of a more aggressive metastatic phenotype [75]. Additionally, capsaicin induced apoptosis in low-grade urothelial cancer but not in high-grade due to the loss of expression of TRV1 [75]. On the other hand, studies performed in pancreatic cells showed that capsaicin apoptosis inducing effects were associated with ROS generation, JNK activation, mitochondrial depolarization, release of cytochrome c in the cytosol and activation of caspase-3 cascade [82]. This results shows that capsaicin is able to activate apoptosis via non-receptor mechanisms [61,82].

As mentioned above, capsaicin induced ROS generation in cancer cells and has been proposed as the principal signaling molecules [69,80]. Induction of apoptosis in cells is associated with significant elevation of intracellular ROS production [39].

The relationship between capsaicin exposure and generation of ROS is also quite complex. In normal cells, ROS are conventionally considered cytotoxic and mutagenic, and at high levels they can induce cell death, apoptosis, and senescence [83]. It has been suggested that capsaicin induces apoptosis in cancerous cells via the generation of even higher levels of intracellular ROS [83].

In recent years, a number of studies have shown that oxidative stress could cause cellular apoptosis via both mitochondria-dependent and mitochondria-independent pathways (Figure 3) [84] In most cells, mitochondria constitute 15%–50% of the total cytoplasmic volume and they participate in metabolic functions, especially those involved in cellular energy production, more than any other organelle. In addition, mitochondria consume approximately 90% of cellular oxygen and are the major source of ROS, which are generated during respiration and involved in maintaining the intracellular redox state [80]. In addition to their long-standing role in energetics, mitochondria represent a point of convergence for many cell death signals in mammalian cells [72]. Interactions at the mitochondrion ultimately determine whether a cell survives or dies in response to many physiologic or therapeutic cell death stimuli. Capsaicin has been shown to target several proteins involved in the mitochondrial death pathway to initiate apoptosis in different cancer cell lines [17,36].

Hail and Lotan [52] found that a 12-h exposure to increasing concentrations of capsaicin was sufficient to promote increasing levels of apoptosis in COLO 16 cells. More than half underwent apoptosis, which was associated with progressive dissipation of mitochondrial transmembrane potential (Δψm) and enhanced superoxide production, reflecting the disintegration of mitochondria and subsequent malfunction of mitochondrial electron transport.

NADPH oxidase constitutes part of complex I of the mitochondrial electron transport chain. It has been found that capsaicin directly inhibits mitochondrial NADPH oxidase activity by binding competitively to the ubiquinone/coenzyme Q site on this enzyme [17]. Therefore, if capsaicin blocks electron transport in mitochondria, dissipation of Δψm should follow. Loss of Δψm is widely understood to initiate apoptosis through causing mitochondrial permeability, which leads to the release of cytochrome c and subsequent activation of pro-apoptotic pathways [52].

A recent study has shown the involvement of mitochondrial Electron Transport Chain (ETC) complexes I and III in capsaicin-induced apoptosis. Pramanik et al. [62] evaluated the mechanism of capsaicin-mediated ROS generation in pancreatic cancer cells. They found that capsaicin reduces complex-I and complex-III activity in BxPC-3 and AsPC-1 cells, leading to ROS generation. Additionally, they also found that the antioxidant levels were lower in capsaicin treated mice tumor cells compared to control, resulting in the accumulation of ROS and mitochondrial damage [62]. Therefore, capsaicin treatment generates ROS through mitochondria and lowers intracellular antioxidants levels, resulting in mitochondrial damage and apoptosis in pancreatic cancer cells [62].

Capsaicin can also inhibit the plasma membrane NADH oxidase by functioning as a coenzyme Q antagonist. The vanillyl moiety of capsaicin is structurally similar to the cyclic portion of coenzyme Q, which could account for the fact that vanilloids act as coenzyme Q antagonists. The inhibition of the plasma membrane NADH oxidase has been reported to be associated with the pro-oxidant and pro-apoptotic properties of capsaicin in certain transformed cells and activated T cells [73].

AMPK interaction is another mechanism proposed by the anticancer activity of capsaicin. AMP-dependent protein kinase (AMPK), considered the principal metabolic gatekeeper of the cell, is a member of a protein kinase family that is activated during ATP-depleting metabolic states, such as hypoxia, heat shock, oxidative stress, and exercise [62]. It functions as a major metabolic switch to maintain energy homeostasis and has been shown to exert as an intrinsic regulator of mammalian cell cycle [85,86]. It is activated by metabolic stresses that increase cellular ADP/ATP and/or AMP/ATP ratios [87]. Activation of AMPK has been related to apoptosis due to metabolic stress and, therefore, AMPK has been proposed as an apoptotic molecule. AMPK activation induced apoptosis in many human cancer cells, and enhanced oxidative stress during apoptosis [85,86].

Upon energetic imbalance, intracellular concentrations of AMP increase, thus promoting AMPK activation. Activated AMPK stimulates catabolic pathways and, concomitantly, inhibits the rate of anabolic reactions to restore the correct adenylate energy charge. It has been shown that capsaicin treatment of colon cancer HT29 cells induced AMPK activation and inhibition of ACC, which is a well-known AMPK substrate, suggesting that capsaicin inhibits lipid biosynthesis. Both phenomena were involved in capsaicin-induced apoptosis [86].

## 7. Capsaicin, Cell Cycle and p53

Cells proliferate through the cell cycle, which is divided into G0/G1, S and G2/M phases. Through the cell cycle, DNA checkpoints exist to ensure the integrity of DNA replication. These checkpoints and repair pathways facilitate cellular responses to DNA damage [36]. Any alteration in these pathways can increase the risk of cancer. Essential parts of the cell-cycle machinery are the cyclins, cyclin-dependent kinases (CDKs) and the CDK inhibitors. Once activated, the CDKs provide a driving force for the cells to move from one phase to the next, but if cyclin and/or CDKs are affected, cell cycle arrest occurs [39,87,88,89]. Chen et al. [88] found that Capsaicin inhibits the proliferation of 5637 bladder carcinoma cells by cycle arrest with the inhibition of CDK2, CDK4 and CDK6.

The tumor suppressor protein p53 regulates the cellular response to DNA damage by mediating cell cycle arrest, DNA repair, and cell death. Phosphorylation at the Ser-15 residue of p53 is critical for p53-dependent transactivation [39]. Capsaicin was found to induce p53 phosphorylation at the Ser-15 residue and enhanced p53 acetylation through down-regulation of sirtuin1, which is responsible for activation of apoptosis [74]. Experiments suggest that p53 is a target of the anticancer mechanism of capsaicin. Arnab et al. [70] incubated human gastric cancer AGS cells with various concentrations of capsaicin in the presence and absence of p53 siRNA. They found that capsaicin induces apoptosis in AGS cells through upregulation of p53 and that the apoptotic activity of capsaicin is p53-dependent. They also found that the ability of capsaicin to induce the expression of pro-apoptotic proteins such as Bax, caspase-3 and caspase-8 was almost completely obliterated by knocking down p53 [70]. Park et al. [90] investigated the effects of capsaicin in the same type of cells and found that the activity of caspase-3 increased with the exposure to capsaicin, suggesting that capsaicin may serve as an anti-tumorigenic agent in human gastric cancer.

Cytokines and chemokines have been shown to play an important role in a number of inflammatory diseases [39]. Several studies have demonstrated that ROS generation plays a significant role in phosphorylation of p53 at the Ser-15 residue [89]. Following capsaicin treatment, an increase in p53 and phosphorylated p53 has been observed in several cancer cell lines [57,77,90,91,92].

## 8. Conclusions

Capsaicinoids and particularly capsaicin are bioactive compounds exhibiting characteristics of great interest to researchers, including for their pharmacobiology applications, such as their recent applications against cancer. Although various *Capsicum* species contain high amounts of capsaicin, not all of these species are used as a source of capsaicin. Therefore, new strategies have been used to increase the content of this secondary metabolite, and thereby generate lines of research that lead from crop care and harvesting to capsaicin purification and application in cancer cell lines. One of these is the use of elicitors, both in crops and plant cell cultures. Further study of the anticancer targets of capsaicin holds potential for novel therapies in the future and warrants more research to improve our understanding of its efficacy in cancer prevention and treatment.

## Figures and Tables

**Table 1:** Studies evaluating the capsaicin effect on cancerous cells.

Cancer Type	Cell Line	Effective Doses (μM)	Anticancer Mechanism	Ref.
Human colorectal cancer	HCT 116	100–200	Induced Autophagy	[40]
	LoVo	100	Induced anti-tumorigenesis. Deregulation of β-catenin/TCF-dependent signaling	[41]
	SW480			[42]
	Colo 205	150	Induced cell death, increased ROS and pro-apoptotic proteins	[43]
Human breast cancer	MCF-7	50–300	Induced Autophagy. Inhibited growth and induced apoptosis	[40,44]
	T47D	200	Inhibited growth, increased apoptosis	[45,46,47,48,49,50]
	BT-474			
	SKBR-3			
	MDA-MB231	20–200	Induced apoptosis and dysfunctions in mitochondria. Antiproliferative activity and arrest of cell cycle into G2/M phase. Enhances the apoptotic effects of TRIAL by activating the calcium-CaMKII-Sp1 pathway	
Human prostate cancer	LNCaP	40–50	Inhibited proliferation. Induced apoptosis	[51,52,53,54,55]
	PC-3	20–50		
	DU-145	500		
	RWPE-1	40		
Human myeloid Leukemia	HL-60	>50	Induced G0/G1 phase cell cycle arrest and apoptosis	[56]
	U937	200	Enhances the apoptotic effects of TRIAL by activating the calcium-CaMKII-Sp1 pathway	[50]
	THP-1			
Human esophageal epidermoid carcinoma	CE 81T/VGH	100	Induced apoptosis and G0/G1 phase cell cycle arrest	[44,49]
Human melanoma	A375	100	Inhibited cell growth and promoted apoptosis	[47,49,57]
Human KB cancer cells	KB cells	150–250	Reduced cell proliferation and viability. Induced cell death and cell cycle arrest in G2/M phase	[58]
Mouse melanoma	B16-F10	50	Inhibited cell migration. Induced apoptosis	[49,59,60]
Human Pancreatic cancer	AsPC-1	150	Inhibited proliferation. Induced apoptosis and generated ROS	[61,62]
	BxPC-3			
	PANC-1	200	Induced G0/G1 phase cell cycle arrest and apoptosis. Inhibited growth	[41,63]
Human multiple myeloma	U266	>5	Inhibited cell proliferation, caused accumulation of cells in G1 phase	[60]
	MM.1S			
Human hepatoma	HepG2	10–200	Decreased cell viability, generated ROS and activated caspase-3. Induced apoptosis and autophagy	[64,65]
	Hep3B	200	Enhances the apoptotic effects of TRIAL by activating the calcium-CaMKII-Sp1 pathway	[50]
Human nasopharyngeal carcinoma	NPC-TW 039	200–400	Induced G0/G1 phase arrest and apoptosis. Increased ROS and activated caspases. Increased cytosolic Ca^2+^	[58]
Human gastric carcinoma	SMC-1	200	Induced apoptosis	[66]
Human bladder cancer	T24	100	Induced ROS production and mitochondrial membrane depolarization	[67]
Human small cell lung cancer	NCI-H69	50	Suppressed growth in all four cell lines	[68]
	NCI-H82			
	DMS53			
	DMS114			

**Table 2:** Studies evaluating capsaicin anticancer effect in vivo.

Animal Model	Capsaicin Doses	Treatment	Results	Ref.
BALB/cJ and BALB/cJ nu/un mice injected with live tumor cells CT26	100–200 μg	Intratumoral on Days 5, 10 and 15	Retarded progression of injected tumors	[81]
BNX nu/un male mice mice injected with PC-3 cells	5 mg/kg/day	Gavage 3 days per week for 4 weeks	Reduced tumor growth	[53]
Athymic nude mice injected with PC-3 cells	5 mg/kg body weight	Subcutaneous injection every two days for 14 days	Suppressed PC-3 tumor growth and induced apoptosis	[51]
Female athymic nude mice injected subcutaneously with AsPC-1 tumor cells	2.5 mg/kg body weight	Five times a week	Suppressed growth of tumor xenografts without adverse effects	[61]
	5 mg/kg body weight	Three times a week		[61]
Male athymic nu/nu mice injected with U266 cells	1 mg/kg	Twice a week for 3 weeks	Inhibited growth of U266 xenograft tumors	[60]
Male Athymic nude mice injected subcutaneously with T24 cells	5 mg/kg	Subcutaneous injection every 3 days for 4 weeks	Slowed growth of xenograph tumors	[67]
Female triple deficient beige/nude/xid mice (BNX) injected with MDA-MB231 cells	5 mg/kg	Oral gavage 3 days per week for 4 weeks	Decrease the size of tumors by 50%	[46]
Male nude mice injected subcutaneously with H69 cells	10 mg/kg body weight	Solid diet until tumors of the control group reached 2000 mm^3^	Tumor growth suppression	[68]
Female BALB/c athymic nude mice injected subcutaneously with Colo 205 cells	1 mg/kg	Intraperitoneal injected. Four weeks of treatment	Inhibition of tumor growth	[46]
	3 mg/kg			
Female athymic nude mice injected subcutaneously with AsPC-1 tumor cells	2.5 mg/kg	Orally fed 5 days a week for 6 weeks	Reduced tumor SOD activity by 60% and increased the ratio of oxidized glutathione to glutathione	[62]
Male BALB/c (nu/nu) athymic nude mice injected subcutaneously with PANC-1 cells	5 mg/kg body weight	Gavage 3 days per week for 4 weeks	Inhibited the growth of pancreatic cancer PANC-1 cell xenografts.	[41]
